# Kisspeptin-10 Improves Testicular Redox Status but Does Not Alter the Unfolded Protein Response (UPR) That Is Downregulated by Hypothyroidism in a Rat Model

**DOI:** 10.3390/ijms25031514

**Published:** 2024-01-26

**Authors:** Luciano Cardoso Santos, Jeane Martinha dos Anjos Cordeiro, Maria Clara da Silva Galrão Cunha, Bianca Reis Santos, Luciana Santos de Oliveira, Adriana Lopes da Silva, Erikles Macêdo Barbosa, Raquel Vieira Niella, Gustavo José Cota de Freitas, Daniel de Assis Santos, Rogéria Serakides, Natália de Melo Ocarino, Stephanie Carvalho Borges, Mário Sérgio Lima de Lavor, Juneo Freitas Silva

**Affiliations:** 1 Electron Microscopy Center, Department of Biological Sciences, State University of Santa Cruz, Campus Soane Nazare de Andrade, Ilheus 45662-900, Brazil; lucianouesc280@gmail.com (L.C.S.); jeane.martinha@gmail.com (J.M.d.A.C.); galrao64@gmail.com (M.C.d.S.G.C.); brsantos_12@hotmail.com (B.R.S.); lubaiucha@gmail.com (L.S.d.O.); doutorado20.24@gmail.com (A.L.d.S.); erikles.mb@gmail.com (E.M.B.); scborges@uesc.br (S.C.B.); 2 Veterinary Hospital, Department of Agricultural and Environmental Sciences, State University of Santa Cruz, Campus Soane Nazare de Andrade, Ilheus 45662-900, Brazil; raquelniella@hotmail.com (R.V.N.); msllavor@uesc.br (M.S.L.d.L.); 3 Department of Microbiology, Institute of Biological Sciences, Federal University of Minas Gerais, Belo Horizonte 31270-901, Brazil; gujcf@yahoo.com.br (G.J.C.d.F.); dasufmg@gmail.com (D.d.A.S.); 4 Department of Veterinary Clinic and Surgery, Veterinary School, Federal University of Minas Gerais, Belo Horizonte 31270-901, Brazil; serakidesufmg@gmail.com (R.S.); nataliaocarino@gmail.com (N.d.M.O.)

**Keywords:** thyroid, male, oxidative stress, reticular stress, rat

## Abstract

Hypothyroidism compromises the testicular redox status and is associated with reduced sperm quality and infertility in men. In this regard, studies have demonstrated the antioxidant potential of kisspeptin in reproductive and metabolic diseases. In this study, we evaluate the effects of kisspeptin-10 (Kp10) on the testicular redox, as well as mediators of the unfolded protein response (UPR) in adult rats with hypothyroidism. Adult male Wistar rats were randomly separated into the Control (*n* = 15), Hypo (*n* = 13) and Hypo + Kp10 (*n* = 14) groups, and hypothyroidism was induced with 6-propyl-2-thiouracil (PTU) for three months. In the last month, half of the hypothyroid animals received Kp10. Testis samples were collected for enzymatic, immunohistochemical and/or gene evaluation of mediators of oxidative stress (TBARs, lipid hydroperoxides (LOOH), ROS, peroxynitrite, SOD, CAT and GPX), endoplasmic reticulum stress (GRP78, ATF6, PERK, CHOP, HO-1 and sXBP1) and antiapoptocytes (BCL-2). Hypothyroidism increased apoptosis index, TBARS and LOOH concentrations, and reduced testicular gene expression of *Sod1*, *Sod2* and *Gpx1*, as well as the expression of *Grp78*, *Atf6*, *Ho1* and *Chop*. Treatment with Kp10, in turn, reduced testicular apoptosis and the production of peroxynitrite, while increased SOD1 and GPX ½ expression, and enzymatic activity of CAT, but did not affect the lower expression of UPR mediators caused by hypothyroidism. This study demonstrated that hypothyroidism causes oxidative stress and dysregulated the UPR pathway in rat testes and that, although Kp10 does not influence the low expression of UPR mediators, it improves the testicular redox status, configuring it as an important antioxidant factor in situations of thyroid dysfunction.

## 1. Introduction

Fertility problems in men can affect up to half of couples trying to have children [1]. This primarily includes endocrine dysfunctions, such as hypothyroidism, which compromises the morphology of the gonads and sexual glands, because it reduces testicular mass [2,3,4,5] and the thickness of the epithelia of the seminiferous tubule [5,6,7], prostate and seminal vesicle [5,7,8]. These effects are possibly the result of steroidogenic failure associated with hypothyroidism, as these glands are highly responsive to testosterone [9,10]. Furthermore, it can result in degenerative and apoptotic changes in the seminiferous epithelium (SE), drastically affecting sperm [5,11,12] and hormonal function [5,7]. 

The changes caused by hypothyroidism in the testicle are also associated with oxidative stress (OS), caused by the high production of reactive oxygen species (ROS), such as superoxide (O_2_^−^), hydroxyl (OH^+^), peroxyl (RO_2_^−^), hydroperoxyl (HO_2_^−^) and hydrogen peroxide (H_2_O_2_) [13,14], or the low production of antioxidant factors such as the enzymes superoxide dismutase (SOD), catalase (CAT) and glutathione peroxidase (GPX) [13].

However, OS can also be associated with other forms of cellular stress, such as endoplasmic reticulum (ER) stress [15], which is characterized by the accumulation of poorly folded proteins in the lumen of the ER [16]. This involves the activation of the unfolded protein response (UPR) pathway [17], mainly by the dissociation of glucose regulatory protein 78 (GRP78) [18,19] from the proteins inositol-requiring enzyme 1 (IRE1), PKR-like ER kinase (PERK) and activating transcription factor 6 (ATF6), which are “ER sensors” [20]. When the cell does not return to its normal functions, an apoptotic pathway is established by activation of the endoplasmic-reticulum-associated protein degradation (ERAD) pathway and the homologous protein C/EBP (CHOP) [17,20]. 

Few studies have been conducted to evaluate ER stress in hypothyroidism; the activation of this process has only been demonstrated in the hypothalamus and maternal-fetal interface of rats [21,22]. In males, ER stress was observed in other disease models such as testicular varicocele [23] and hypercholesterolemia [24], and in models of cadmium-induced cellular toxicity [25] and fluorine [26], but it is unknown whether hypothyroidism causes ER stress in the testes. 

Studies have been conducted to evaluate the potential of substances to reduce the effects of OS and/or ER stress in the reproductive tract [27,28]. In this regard, kisspeptin, which control the hypothalamic release of gonadotropin-releasing hormone (GnRH) [29,30] and has local action in the testes [31,32], has been recognized for its antioxidant effects in models of ovarian and uterine, [33] testicular, [34], cardiac [35] and brain disease in mice [36]. In addition, in vitro overexpression of kisspeptin in human granulosa cells increased proliferation, inhibited apoptosis, suppressed ROS generation, reduced malondialdehyde (MDA) levels, and increased levels of antioxidant factors [37]. These effects observed in several studies justify the use of kisspeptin as a potent antioxidant factor in disease models.

We recently demonstrated that kisspeptin blocks OS and reduces the expression of ER stress mediators in the placenta of hypothyroid rats [27]. Furthermore, in males, it reestablished the height of the seminiferous epithelium, tubular diameter, testosterone production and sperm quality of hypothyroid rats [5]. Therefore, the two main hypotheses of this study are that hypothyroidism causes oxidative and ER stress, and that kisspeptin-10 (Kp10) treatment blocks these cellular stresses in the adult rat testis. Our results demonstrated that Kp10 improves testicular antioxidant defense because it increased the protein expression of SOD and GPX, reducing the percentage of tubules undergoing apoptosis but not influencing the low expression of UPR mediators in the testis of rats with hypothyroidism.

## 2. Results

### 2.1. Confirmation of Hypothyroidism

Hypothyroidism was confirmed by reduced body mass gain and reduced levels of free T_4_ (Table 1).

### 2.2. Immunolocalization of 8-OhdG and Quantification of LOOH, TBARS, ROS and Peroxynitrite

**Figure 1:**
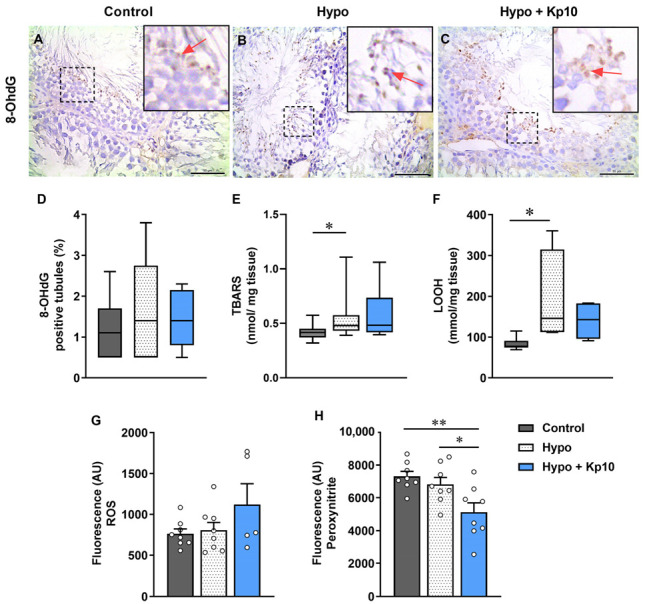
Effects of hypothyroidism and Kp10 treatment in 8-OhdG staining and quantification of TBARS, LOOH, ROS and peroxynitrite in the rat testes. (**A**–**C**) Photomicrographs of 8-OhdG staining (red arrows) in the testes of rats from Control (**A**), Hypo (**B**) and Hypo + Kp10 (**C**) groups; Hematoxylin; Bar = 50 µm. (**D**) Percentage of seminiferous tubules positive for 8-OhdG in the rat testes (*n* = 5–6). (**E**,**F**) Tissue concentration of TBARS (**E**), LOOH (**F**), ROS (**G**) and peroxynitrite (**H**) in the testes of rats from Control, Hypo and Hypo + Kp10 groups (*n* = 5–8). Legends: 8-OhdG = 8-hydroxyl–2′–deoxyguanosine; TBARS = thiobarbituric acid-reactive substances; LOOH = Lipid Hydroperoxides; ROS = reactive oxygen species; * *p* < 0.05; ** *p* < 0.01.

Immunolabeling of 8-hydroxyl–2′–deoxyguanosine (8-OhdG) was restricted to round and/or elongated spermatids (red arrows) in stages VI or VII tubules (Figure 1A–C). However, the percentage of labeled tubules did not differ between the groups (Figure 1D; *p* > 0.05). The amount of testicular thiobarbituric acid-reactive substances (TBARS) (Figure 1E) and lipid hydroperoxides (LOOH) (Figure 1F) was higher in the hypothyroid (Hypo) group, confirming the oxidative stress. The total ROS in testis was similar between groups (Figure 1G; *p* > 0.05) and no differences in peroxynitrite were observed between the control (Control) and Hypo group (Figure 1H; *p* > 0.05). However, when treated with Kp10 (Hypo + Kp10), the rats had lower amounts of peroxynitrite, even in relation to the Control group (Figure 1H).

### 2.3. Treatment with Kp10 Increases Protein Expression of SOD1 and GPX1/2 and Catalase Enzyme Activity in the Testes of Hypothyroid Rats

**Figure 2:**
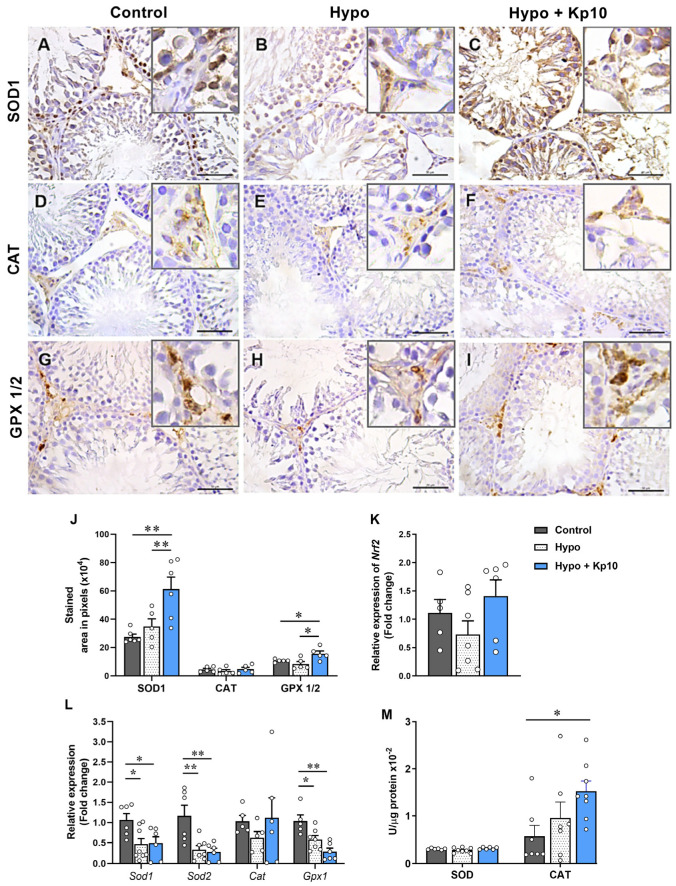
Effects of hypothyroidism and Kp10 treatment on the expression of antioxidant mediators in rat testes. (**A**–**I**) Photomicrographs of SOD1 (**A**–**C**), CAT (**D**–**F**), GPX (**G**–**I**) staining in the testes of rats of the Control (**A**,**D**,**G**), Hypo (**B**,**E**,**H**) and Hypo + Kp10 (**C**,**F**,I) groups; hematoxylin; bar = 50 µm; highlights show interstitial immunostaining of proteins. (**J**) Stained area of SOD1, CAT and GPX 1/2 in the rat testes (*n* = 5–6). (**K**) Relative gene expression of *Nrf2* (*n* = 5–7). (**L**) Relative gene expression of *Sod1*, *Sod2*, *Cat*, and *Gpx1* (*n* = 5–8). (**M**) Enzyme activity of SOD and CAT (*n* = 5–8). Legends: SOD 1 = superoxide dismutase 1; CAT = catalase; GPX ½ = glutathione peroxidase 1/2; *Nrf2* = gene encoding nuclear factor erythroid 2-related factor 2; *Sod1* = gene encoding SOD1; *Sod2* = gene encoding SOD 2; *Cat* = gene encoding CAT; *Gpx1* = gene encoding GPX 1; * *p* < 0.05; ** *p* < 0.01.

The gene expression of *Nrf2*, important transcription factor involved in the expression of antioxidant enzymes under hypoxic conditions [38], showed similar mRNA levels between the control, hypothyroid and Kp10-treated animals (Figure 2K; *p* > 0.05). However, in the Hypo + Kp10 group, SE and interstitial cells showed more intense staining of SOD1 and GPX ½, respectively, when compared to the Control and Hypo groups (Figure 2A–C,G–I), which was confirmed by analyzing the immunostaining area (Figure 2J). Although no difference was observed in the enzymatic activity of SOD between the groups (Figure 2M; *p* > 0.05), the gene expression of *Sod1*, *Sod2* and *Gpx1* was reduced in the testes of rats with hypothyroidism (Figure 2L), while treatment with Kp10 did not alter this low expression (*p* > 0.05).

Regarding CAT, no significant difference was observed in the immunostaining area and gene expression between groups (Figure 2D–F,J,L; *p* > 0.05). However, catalase enzyme activity was higher in the testes of Kp10-treated hypo rats (Figure 2M). 

### 2.4. Kp10 Treatment Does Not Alter Lower Testicular Gene Expression of UPR Mediators Caused by Hypothyroidism in Rats

**Figure 3:**
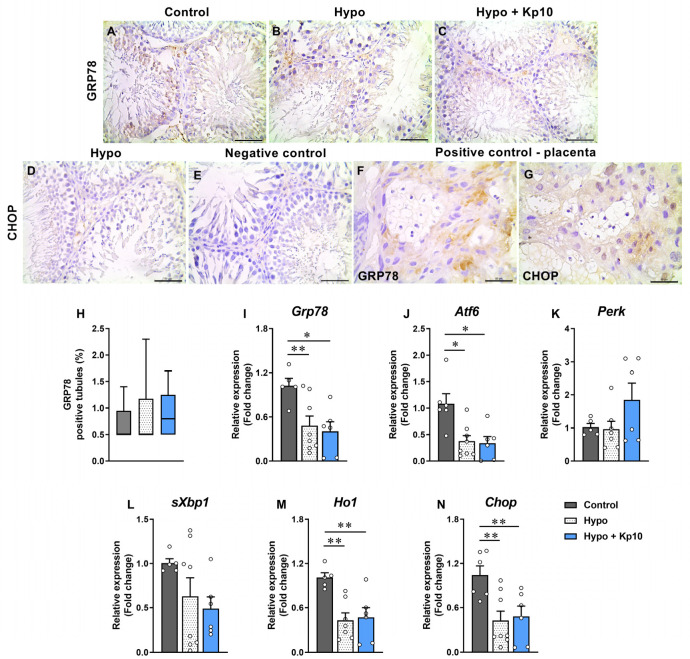
Effects of hypothyroidism and Kp10 treatment on the expression of UPR mediators and ER stress in rat testes. (**A**–**G**) Photomicrographs of GRP78 (**A**–**C**) and CHOP (**D**) immunolabeling in the testes of rats from Control (**A**), Hypo (**B**,**D**) and Hypo + Kp10 (**C**) groups; hematoxylin; bar = 50 µm. (**E**–**G**) Photomicrographs of negative (**E**) and positive controls (hypothyroid rat placenta) for GRP78 (**F**) and CHOP (**G**). (**H**) Percentage of seminiferous tubules positive for GRP78 in the rat testes. (**I**–**N**) Relative gene expression of *Grp78* (**I**), *Atf6* (**J**), *Perk* (**K**), *sXbp1* (**L**), *Ho1* (**M**) and *Chop* (**N**) in the rat testes (*n* = 5–8). Legends: GRP78 = Heat shock protein family A (Hsp70) member 5; CHOP = homologous protein C/EBP; *Grp78* = gene encoding GRP78; *Chop* = gene encoding CHOP; *Atf6* = gene encoding Activating transcription factor 6, *Perk* = gene encoding Eukaryotic translation initiation factor 2 alpha kinase 3; *sXbp1* = gene encoding X-box binding protein 1; *Ho1* = gene enconding Heme oxygenase 1; * *p* < 0.05, ** *p* < 0.01.

The GRP78 protein showed weak staining in the cytoplasm of SE cells and no significant difference was observed in the number of stained tubules between the groups (Figure 3A–C,H; *p* > 0.05). In contrast, no CHOP labeling was observed in the SE and in the interstitium (Figure 3D). Interestingly, regarding gene expression, a significant reduction was observed in the expression of *Grp78* (Figure 3I), *Atf6* (Figure 3J), *Ho1* (Figure 3M) and *Chop* (Figure 3N) in the Hypo group compared to the Control; treatment with Kp10 did not alter this lower expression (*p* > 0.05). For genes *Perk* (Figure 3K) and *sXbp1* (Figure 3L), no difference was observed between the groups (*p* > 0.05).

### 2.5. Kp10 Treatment Reduces the Apoptotic Index in Testicular Cells

**Figure 4:**
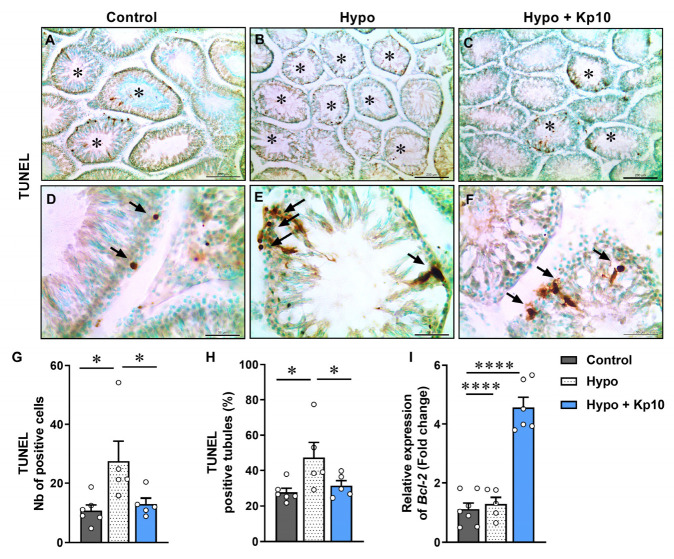
Effects of hypothyroidism and Kp10 treatment on the apoptosis index in rat testes. (**A**–**F**) Photomicrographs of TUNEL in the testes of rats from Control (**A**,**D**), Hypo (**B**,**E**) and Hypo + Kp10 (**C**,**F**) groups; arrows indicate apoptosis cells in seminiferous epithelium; asterisk indicate TUNEL positive tubules; methyl green; bar = 200 µm (**A**–**C**) and 50 µm (**D**–**F**). (**G**) Total number of TUNEL positive cells in the rat testes. (**H**) Percentage of TUNEL positive tubules in the rat testes (*n* = 5–6). (**I**) Relative gene expression of *Bcl-2* in rat testes (*n* = 5–7). Legends: *Bcl-2* = gene encoding Apoptosis regulator BCL-2; * *p* < 0.05; **** *p* < 0.0001.

The number of apoptotic cells and the percentage of tubules presenting apoptotic nuclei were significantly increased in rats from the Hypo group (Figure 4B,E,G–H). On the other hand, animals treated with Kp10 showed a reduction in this percentage, matching the Control (Figure 4G–H), accompanied by a significant increase in the expression of *Bcl-2* mRNA, an important anti-apoptotic factor (Figure 4I) [39].

## 3. Discussion

The kisspeptin/kiss1r system is known to regulate the release of GnRH and luteinizing hormone (LH) in the hypothalamic-pituitary-gonadal (HPG) axis, but it is also known for local functions in the testis (e.g., in testis formation, spermatogenesis and testicular steroidogenesis [31,32]). In this study, we demonstrated that Kp10 improves testicular antioxidant status and reduces apoptosis in adult male rats with hypothyroidism, although it is not able to improve testicular dysregulation of mediators of UPR pathway.

OS is one of the main factors associated with reproductive dysfunction in males [28]. To verify oxidative damage in the testis of rats with hypothyroidism or treated with Kp10, we initially evaluated the presence of 8-OhdG, a biomarker of DNA oxidation [40], along with TBARS, LOOH, ROS and peroxynitrite concentration. The count of 8-OhdG-positive tubules did not differ between groups, and staining was mainly in spermatids in tubules at stages VII–VIII. This is similar to what was observed by Feng et al. [41] in a fluoride-induced OS model, in which 8-OhdG labeling occurred in elongated spermatids. Although hypothyroidism did not increase oxidative DNA damage, the observed increase in TBARS and LOOH concentrations confirms OS status. This is consistent with other studies that have described an increase in lipid peroxidation in the testis of hypothyroid animals [42,43].

Kp10, despite not having significant effects on increasing TBARS and LOOH levels, reduced peroxynitrite, an important reactive nitrogen species [44]. Interestingly, this occurred in parallel with the increase in the activity of the CAT enzyme in this group. Studies have already demonstrated that CAT can be inhibited by nitric oxide (NO) and peroxynitrite [45,46], and that CAT itself is also capable of conducting the oxidation of NO and the decomposition of peroxynitrite. A previous study with gastric carcinoma cells demonstrated that inhibition of CAT allowed selective reactivation of the NO/peroxynitrite pathway [47], thus revealing the role of this enzyme in the catabolism of this factor. Taken together, our data suggest that the increase in CAT activity caused by Kp10 may have favored the reduction of peroxynitrite in the testes and suggest the participation of this peptide in the testicular regulation of the NO/peroxynitrite pathway.

In addition to CAT, we evaluated the expression and/or activity profile of other enzymes, such as SOD and GPX. The genes *Sod1*, *Sod2* and *Gpx1* were reduced in tests on rats with hypothyroidism, which is similar to previous studies conducted on this species [42,43,48]. Although Kp10 did not change the low expression of these genes caused by hypothyroidism, a significant increase in the immunostaining of SOD1 and GPX1/2 was observed after treatment, highlighting their antioxidant effects in tests on hypothyroid rats. This corroborates the findings of previous studies in which the administration of Kp10 increased antioxidant defense at the maternal-fetal interface of hypothyroid rats [27], as also observed in models of ovarian and uterine [33], testicular [34], cardiac [35] and brain dysfunctions in mice [36]. The increase in antioxidant defense caused by Kp10 in the present study may be involved in the improvement of testicular morphology and steroidogenesis and sperm quality observed in hypothyroid rats after treatment with Kp10 [5].

In addition to OS, we evaluated the expression of several factors involved in the UPR pathway and ER stress in the testes of rats with hypothyroidism. Except for the genes *sXbp1* and *Perk*, all the other analyzed genes (*Grp78*, *Atf6*, *Ho1*, *Chop*) showed reduced expression in hypothyroid animals, while treatment with Kp10 did not alter this low expression. This shows that hypothyroidism does not activate the UPR pathway in the rat testis as occurs in other experimental models, but deregulates it, which can be better understood as a “failure in the endoplasmic reticulum stress response” [49]. This has also been observed in the maternal-fetal interface of hypothyroid rats at 14 days of pregnancy [22] and in studies involving obesity and aging [49], but is critical for the cell because the UPR pathway also helps maintain its viability due to its involvement in protein synthesis [50]. 

The low GRP78 expression can alter several cellular functions given its activity in the proper folding of polypeptides or degradation of poorly folded products, in the transport of membrane or secretory proteins, and even in the intracellular homeostasis of calcium ions (Ca^2+^) [51]. In fact, hypothyroidism in male rats is known to reduce the testicular concentration of Ca^2+^ [43] and the activity of Ca^2+^—ATPase [48], which is an important enzyme involved in intracellular Ca ^2+^ balance. Impaired calcium balance, in turn, can lead to mitochondrial and testicular dysfunction [52,53]. In the same way, ATF6 is also a critical factor associated with the development and homeostasis of various organs [54] and fails in its expression has been associated with reduced fertility in male mice [55].

In addition to GRP78 and ATF6, the dysregulation of HO-1 observed in the testes of hypothyroid rats may be critical for the function of this organ. HO-1 is well known for its role in regulating OS [13,14]. However, human studies and experimental models of HO-1 deficiency have shown that this enzyme is involved in controlling several other bodily functions [56], including anti-inflammatory properties [57], iron control [58], and in glucose metabolism and mitochondrial respiration [59]. In the testes, the administration of hemin, an HO-1inducer, improved testicular steroidogenesis, sperm quality, and the synthesis of sex hormones and reduced DNA fragmentation [60].

Although activation of the UPR pathway and ER stress were not observed in this study, a significant increase in the testicular apoptosis in the hypothyroid rats was observed in other previous studies involving thyroid hypofunction [43,61]. Surprisingly, animals that received Kp10 showed a lower amount of apoptosis in testicular cells. To understand its possible action in this pathway, the BCL-2 factor was evaluated. In fact, the reduction in testicular apoptosis appears to be via BCL-2, which showed gene expression almost three times higher in this group. Studies involving kisspeptin and apoptosis are inconsistent, but some have already demonstrated roles in attenuating apoptosis in neurons [62] and in granulosa cells in rat polycystic ovary model [37], which also showed an increase in BCL-2 [37,62], considered an antiapoptotic factor [39].

## 4. Materials and Methods

### 4.1. Animals and Experimental Design

Two-month-old male Wistar rats were divided into three groups: Control (*n* = 15), Hypo (*n* = 13) and Hypo + Kp10 (*n* = 14; 12 µg/Kg/day; Cat. Nb. 4243, Tocris Bioscience, Bristol, UK). Hypothyroidism was induced by oral administration of 6-propyl-2-thiouracil (PTU; 4 mg/kg/day; Sigma-Aldrich, St. Louis, MO, USA) diluted in 3 mL of distilled water for 3 months, while the control group received the same volume of water as placebo. Body mass was monitored throughout the experiment, and blood was collected for free T_4_ measurement. The experiments were conducted as previously described by Santos et al. [5] (CEUA 03/19).

### 4.2. Immunohistochemistry (IHC)

The antibodies used were anti-8-OhdG (1:50; sc-393871), anti-SOD1 (1:5000, sc-101523), anti-CAT (1:200, sc-271803), anti-GPX1/2 (1:500, sc-133160), anti-GRP78 (1:50, sc-13539) and anti-CHOP (1:50, sc-71136), from Santa Cruz Biotechnology, CA, USA. The indirect streptavidin-biotin-peroxidase method was used with the Dako detection system (EnVision FLEX+, Mouse, High pH, (Link); Dako North America, Inc., CA, USA) following the protocol of Ilie et al. [63] and adaptations of Santos et al. [5]. The sections were counterstained with Harris hematoxylin and the negative control was obtained by replacing the primary antibody with TBS-T. The placenta of rats with hypothyroidism was used as a positive control [22]. 

Descriptive and quantitative analyses of SOD1, CAT, GPX1/2, 8-OHdG and GRP78 immunolabeling were performed in the seminiferous tubules and interstitium. The immunolabeling area was defined using WCIF ImageJ software version 1.41 (Media Cybernetics Manufacturing, Rockville, MD, USA) on random photomicrographs taken on 10–15 regions of the testes under a Leica DM 2500 microscope using the Leica DFC 295 digital camera (Leica Microsystems, Wetzlar, Germany). For analysis, color deconvolution and thresholding of the images were performed. The data of each tissue were expressed as immunolabeling area in pixels [64]. 

### 4.3. RNA Extraction and Real-Time Quantitative PCR (qPCR)

Total RNA extraction from the testes was performed using TRizol (Invitrogen, Life Technologies, Carlsbad, CA, USA), and the cDNA was synthesized as previously described [5]. Primers were designed based on the *Rattus norvegicus* mRNA sequence (Table 2) and the relative gene expression was calculated using the 2^−∆∆CT^ method [65], in which the change in the expression of target genes in samples from the Hypo and Hypo + Kp10 groups in relation to the Control group was obtained by the difference between ΔCT (CT = Cycle Threshold) of the target genes by the ΔCT of the reference gene in base 2. For this study, *Gapdh* was used as a normalizing gene.

### 4.4. Enzymatic Activity of Superoxide Dismutase (SOD) and Catalase (CAT)

The crude extract of the testes samples was obtained by homogenization with 50 nmoL of potassium phosphate buffer (PPB) (pH 7.0), followed by sonication under 70% amplitude with 8 pulses of 5 s and intervals of 10 s, totaling 40 s. Then, the samples were centrifuged at 13,400× *g* rpm at 4 °C for 10 min to collect the supernatants. The protein concentration was determined by the Bradford method [66], and enzymatic activities of SOD and catalase were evaluated according to Marklund and Marklund [67] and Aebi [68], respectively.

### 4.5. Lipid Peroxidation 

Lipid peroxidation was assessed using MDA concentrations in the thiobarbituric acid reaction (TBARS) and through the levels of lipid hydroperoxides (LOOH). Quantification of TBARS was performed as described by Oliveira et al. [69]. Briefly, 200 μL testicular sample supernatant was incubated with 500 μL of thiobarbituric acid (TBA, 0.8%; pH 3.2), 500 μL of acetic acid buffer, 200 μL of sodium dodecyl sulfate (SDS; 8.1 %; Invitrogen Life Technologies, Carlsbad, CA, USA), and 100 μL water. The Eppendorf’s were sealed and incubated for 2 h on a hot plate at 95 °C. After that, they were cooled on ice and centrifuged for 10 min at 1200× *g* rpm. Next, 1 mL was carefully transferred to a quartz cuvette and the absorbance was measured in a spectrophotometer at 532 nm. A standard curve of Malondialdehyde potassium salt enolate (Ref. SMB00976; Sigma-Aldrich, SP, Brazil) was used at concentrations of 1, 1.5, 3, 6 and 9 nmol and the mean calibration factor (MCF) was obtained. TBARS values were estimated by multiplying the absorbances obtained by MCF and the results expressed as nmol of TBARS per mg of tissue.

Quantification of LOOH was performed as described by Borges et al. [70]. The supernatant of testicular samples was homogenized in 90% methanol (Synth; SP, Brazil) and centrifuged at 10,000× *g* for 30 min at 4 °C. The supernatant and reaction medium composed of 90% methanol, xylenol orange (Ref. 398187; Sigma-Aldrich, SP, Brazil), 25 mM sulfuric acid (H_2_SO_4_; Ref. A1060; Synth; SP, Brazil), 4 mM butylated hydroxytoluene (BHT; Ref. 1082708; Sigma-Aldrich, SP, Brazil) and 250 mM FeSO_4_NH_4_ were incubated for 30 min at 25 °C in microplates. Reading was performed at 560 nm and LOOH concentration determined based on an extinction coefficient of 4.3 mM cm^−1^ and is expressed as mmol LOOH mg^−1^ of tissue.

### 4.6. Reactive Oxygen Species (ROS) and Peroxynitrite

ROS and peroxynitrite were measured in testicular samples as performed by Dos Anjos Cordeiro [22]. Endogenous amounts of ROS and peroxynitrite were measured in testes samples by fluorometric assay with specific probes for ROS (dichlorofluorescein 2′,7′-diacetate; DCFH-DA, Invitrogen, Life Technologies, Carlsbad, CA, USA) and peroxynitrite (dihydrorhodamine 123, Invitrogen, Life Technologies, Carlsbad, CA, USA) [71]. Fluorescence was measured with a fluorometer (Synergy 2 SL Luminescence Microplate Reader; Biotek^®^ Instruments, Inc.; Winooski, VT, USA) using excitation and emission wavelengths of 485–525 nm, respectively. Data were expressed as arbitrary units (AU) of fluorescence + SEM. These assays were performed in duplicate.

### 4.7. TUNEL Assay

Apoptotic cells in the testicular samples were evaluated using an apoptosis detection kit (TdT-FragEL DNA Fragmentation Detection Kit, Calbiochem, San Diego, CA, USA) according Silva et al. [72]. Positive cells or tubules were counted in 10 histological sections per animal photographed randomly under a 10× objective lens (100× total magnification). On average, 100 to 120 tubules were counted per animal.

### 4.8. Statistical Analysis

The data were represented by mean ± S.E.M or median with maximum and minimum limits. Analysis of variance (ANOVA) was performed, followed by the Student–Newman-Keuls test (SNK) or Kruskal–Wallis test using GraphPad Prism 8.0.2 software. The differences were considered significant if *p <* 0.05. 

## 5. Conclusions

The findings of this study characterized the redox status and highlighted, for the first time, the dysregulation of UPR mediators in the testes of adult rats associated with hypothyroidism. Although Kp10 treatment did not influence the low expression of UPR mediators, it was sufficient to increase testicular antioxidant defenses in these animals and reduce apoptosis in testicular cells. Therefore, we suggest that kisspeptin analogues are promising antioxidants in the treatment of testicular dysfunction caused by thyroid hypofunction.

## Figures and Tables

**Table 1:** Body mass and plasma concentration of free T_4_ in rats from the control, hypothyroid and hypothyroid treated with kisspeptin-10 groups.

Parameter	Control	Hypothyroid	Hypothyroid + Kp10
Initial body mass (g)	370.7 ± 6.20	371.7 ± 6.27	371.7 ± 7.68
Final body mass (g)	423.1 ± 5.87	345.0 ± 6.55 ****	333.5 ± 8.07 ****
Free T_4_ (ng/dL)	1.699 ± 0.097	0.029 ± 0.014 ****	0.041 ± 0.016 ****

Legends: **** *p* < 0.0001. (Mean ± SEM); *n* = 13–15 animals/group.

**Table 2:** List of primer pairs for RT-qPCR.

Gene		Sequences (5′ → 3′)	Accession No.
Superoxide dismutase 1	*Sod1*	GAAAGGACGGTGTGGCCAAT	NM_017050.1
		CTCGTGGACCACCATAGTACG	
Superoxide dismutase 2	*Sod2*	CGGGGGCCATATCAATCACA	NM_017051.2
		GCCTCCAGCAACTCTCCTTT	
Catalase	*Cat*	CTGACTGACGCGATTGCCTA	NM_012520.2
		GTGGTCAGGACATCGGGTTT	
Glutathione peroxidase 1	*Gpx1*	GCGCTACAGCGGATTTTTGA	NM_030826.4
		GAAGGCATACACGGTGGACT	
Nuclear factor, erythroid 2-like 2	*Nrf2*	CCCATTGAGGGCTGTGATCT	NM_031789.2
		GCCTTCAGTGTGCTTCTGGTT	
Heat shock protein family A (Hsp70) member 5	*Grp78*	TGAAGGGGAGCGTCTGATTG	NM_013083.2
		TCATTCCAAGTGCGTCCGAT	
Activating transcription factor 6,	*Atf6*	CCAGCAGAAAACCCGCATTC	XM_017598829.1
		CAGAATTCCTGATGCTAGTGGTT	
Eukaryotic translation initiation factor 2 alpha kinase 3	*Perk*	GGCTGGTGAGGGATGGTAAA	NM_031599.2
		TTGGCTGTGTAACTTGTGTCATCA	
X-box binding protein 1	*sXbp1*	CTGAGTCCGCAGCAGGTG	NM_001271731.1
		AAGAGGCAACAGCGTCAGAA	
Heme oxygenase 1	*Ho1*	ACAGCACTACGTAAAGCGTCTCCA	NM_012580.2
		CATGGCCTTCTGCGCAATCTTCTT	
Apoptosis regulator BCL-2	*Bcl-2*	ACTTCTCTCGTCGCTACCGTC	NM_016993.2
		AAGAGTTCCTCCACCACCGT	
Glyceraldehyde-3-Phosphate dehydrogenase	*Gapdh*	ACAGCCGCATCTTCTTGTGC	NM_017008.4
		GCCTCACCCCATTTGATGTT	

## Data Availability

Data will be made available on request.

## References

[1] Leslie S.W., Soon-Sutton T.L., Khan M.A. Male Infertility. https://www.statpearls.com/articlelibrary/viewarticle/23503/.

[2] Sengupta P., Dutta S., Karkada I.R., Chinni S.V. (2022). Endocrinopathies and Male Infertility. Life.

[3] Alahmar A., Dutta S., Sengupta P. (2019). Thyroid Hormones in Male Reproduction and Infertility. Asian Pac. J. Reprod..

[4] Wagner M.S., Wajner S.M., Maia A.L. (2008). The Role of Thyroid Hormone in Testicular Development and Function. J. Endocrinol..

[5] Santos L.C., Martinha J., Santana S., Barbosa E.M., Santos B.R., Mendonça L.D., Clara M., Cunha G., Machado W.M., Santana L.R. (2023). Kisspeptin Treatment Reverses High Prolactin Levels and Improves Gonadal Function in Hypothyroid Male Rats. Sci. Rep..

[6] Ibrahim A.A., Mohammed N.A., Eid K.A., Abomughaid M.M., Abdelazim A.M., Aboregela A.M. (2021). Hypothyroidism: Morphological and Metabolic Changes in the Testis of Adult Albino Rat and the Amelioration by Alpha-Lipoic Acid. Folia Morphol..

[7] François Xavier K.N., Patrick Brice D.D., Modeste W.N., Esther N., Albert K., Pierre K., Pierre W. (2019). Preventive Effects of Aframomum Melegueta Extracts on the Reproductive Complications of Propylthiouracil-Induced Hypothyroidism in Male Rat. Andrologia.

[8] Sahoo D.K., Roy A. (2012). Compromised Rat Testicular Antioxidant Defence System by Hypothyroidism before Puberty. Int. J. Endocrinol..

[9] Mann T. (1974). Secretory Function of the Prostate, Seminal Vesicle and Other Male Accessory Organs of Reproduction. J. Reprod. Fertil..

[10] Higgins S.J., Burchell J.M. (1978). Effects of Testosterone on Messenger Ribonucleic Acid and Protein Synthesis in Rat Seminal Vesicle. Biochem. J..

[11] Alipour F., Jalali M., Nikravesh M.R., Fazel A., Sankian M., Khordad E. (2018). Assessment of Sperm Morphology, Chromatin Integrity, and CatSper Genes Expression in Hypothyroid Mice. Acta Biol. Hung..

[12] Romano R.M., Gomes S.N., Cardoso N.C.S., Schiessl L., Romano M.A., Oliveira C.A. (2017). New Insights for Male Infertility Revealed by Alterations in Spermatic Function and Differential Testicular Expression of Thyroid-Related Genes. Endocrine.

[13] Bolisetty S., Jaimes E. (2013). Mitochondria and Reactive Oxygen Species: Physiology and Pathophysiology. Int. J. Mol. Sci..

[14] Bayr H. (2005). Reactive Oxygen Species. Crit. Care Med..

[15] Burton G.J., Yung H.W. (2011). Endoplasmic Reticulum Stress in the Pathogenesis of Early-Onset Pre-Eclampsia. Pregnancy Hypertens.

[16] Zhang K., Kaufman R.J. (2008). From Endoplasmic-Reticulum Stress to the Inflammatory Response. Nature.

[17] Almanza A., Carlesso A., Chintha C., Creedican S., Doultsinos D., Leuzzi B., Luís A., McCarthy N., Montibeller L., More S. (2019). Endoplasmic Reticulum Stress Signalling—From Basic Mechanisms to Clinical Applications. FEBS J..

[18] Szegezdi E., Logue S.E., Gorman A.M., Samali A. (2006). Mediators of Endoplasmic Reticulum Stress-Induced Apoptosis. EMBO Rep..

[19] Ibrahim I.M., Abdelmalek D.H., Elfiky A.A. (2019). GRP78: A Cell’s Response to Stress. Life Sci..

[20] Veerbeek J.H.W., Tissot Van Patot M.C., Burton G.J., Yung H.W. (2015). Endoplasmic Reticulum Stress Is Induced in the Human Placenta during Labour. Placenta.

[21] Torres-Manzo A.P., Franco-Colín M., Blas-Valdivia V., Pineda-Reynoso M., Cano-Europa E. (2018). Hypothyroidism Causes Endoplasmic Reticulum Stress in Adult Rat Hippocampus: A Mechanism Associated with Hippocampal Damage. Oxid. Med. Cell. Longev..

[22] dos Anjos Cordeiro J.M., Santos L.C., de Oliveira L.S., Santos B.R., Santos E.O., Barbosa E.M., de Macêdo I.O., de Freitas G.J.C., de Assis Santos D., de Lavor M.S.L. (2022). Maternal Hypothyroidism Causes Oxidative Stress and Endoplasmic Reticulum Stress in the Maternal-Fetal Interface of Rats. Free Radic. Biol. Med..

[23] Hosseini M., Shaygannia E., Rahmani M., Eskandari A., Golsefid A.A., Tavalaee M., Gharagozloo P., Drevet J.R., Nasr-Esfahani M.H. (2020). Endoplasmic Reticulum Stress (ER Stress) and Unfolded Protein Response (UPR) Occur in a Rat Varicocele Testis Model. Oxid. Med. Cell. Longev..

[24] Yu C., Jiang F., Zhang M., Luo D., Shao S., Zhao J., Gao L., Zuo C., Guan Q. (2019). HC Diet Inhibited Testosterone Synthesis by Activating Endoplasmic Reticulum Stress in Testicular Leydig Cells. J. Cell. Mol. Med..

[25] Ji Y.L., Wang H., Zhang C., Zhang Y., Zhao M., Chen Y.H., Xu D.X. (2013). N-Acetylcysteine Protects against Cadmium-Induced Germ Cell Apoptosis by Inhibiting Endoplasmic Reticulum Stress in Testes. Asian J. Androl..

[26] Zhang S., Jiang C., Liu H., Guan Z., Zeng Q., Zhang C., Lei R., Xia T., Gao H., Yang L. (2013). Fluoride-Elicited Developmental Testicular Toxicity in Rats: Roles of Endoplasmic Reticulum Stress and Inflammatory Response. Toxicol. Appl. Pharmacol..

[27] Santos B.R., dos Anjos Cordeiro J.M., Santos L.C., Barbosa E.M., Mendonça L.D., Santos E.O., de Macedo I.O., de Lavor M.S.L., Szawka R.E., Serakides R. (2022). Kisspeptin Treatment Improves Fetal-Placental Development and Blocks Placental Oxidative Damage Caused by Maternal Hypothyroidism in an Experimental Rat Model. Front. Endocrinol..

[28] Asadi N., Bahmani M., Kheradmand A., Rafieian-Kopaei M. (2017). The Impact of Oxidative Stress on Testicular Function and the Role of Antioxidants in Improving It: A Review. J. Clin. Diagn. Res..

[29] Gottsch M.L., Cunningham M.J., Smith J.T., Popa S.M., Acohido B.V., Crowley W.F., Seminara S., Clifton D.K., Steiner R.A. (2004). A Role for Kisspeptins in the Regulation of Gonadotropin Secretion in the Mouse. Endocrinology.

[30] Pinilla L., Aguilar E., Dieguez C., Millar R.P., Tena-Sempere M. (2012). Kisspeptins and Reproduction: Physiological Roles and Regulatory Mechanisms. Physiol. Rev..

[31] Cao Y., Li Z., Jiang W., Ling Y., Kuang H. (2019). Reproductive Functions of Kisspeptin/KISS1R Systems in the Periphery. Reprod. Biol. Endocrinol..

[32] Feng T., Bai J.H., Xu X.L., Liu Y. (2019). Kisspeptin and Its Effect on Mammalian Spermatogensis. Curr. Drug Metab..

[33] Aslan M., Erkanli Senturk G., Akkaya H., Sahin S., Yılmaz B. (2017). The Effect of Oxytocin and Kisspeptin-10 in Ovary and Uterus of Ischemia-Reperfusion Injured Rats. Taiwan J. Obstet. Gynecol..

[34] Akkaya H., Eyuboglu S., Erkanlı Senturk G., Yilmaz B. (2017). Investigation of the Effects of Kisspeptin-10 in Methionine-Induced Lipid Peroxidation in Testicle Tissue of Young Rats. J. Biochem. Mol. Toxicol..

[35] Akkaya H. (2021). Kisspeptin-10 Administration Regulates MTOR and AKT Activities and Oxidative Stress in Mouse Cardiac Tissue. J. Evol. Biochem. Physiol..

[36] Akkaya H., Sümer E., Kutlu S., Solak H., Yilmaz B. (2022). What Is the Protective Effect of Preischemic Kisspeptin-10 Administration against Ischemia/Reperfusion Injury of Striatum on Mice?. Turk. J. Med. Sci..

[37] Sun P., Zhang Y., Sun L., Sun N., Wang J., Ma H. (2023). Kisspeptin Regulates the Proliferation and Apoptosis of Ovary Granulosa Cells in Polycystic Ovary Syndrome by Modulating the PI3K/AKT/ERK Signalling Pathway. BMC Womens Health.

[38] Francisqueti-Ferron F.V., Ferron A.J.T., Garcia J.L., de Almeida Silva C.C.V., Costa M.R., Gregolin C.S., Moreto F., Ferreira A.L.A., Minatel I.O., Correa C.R. (2019). Basic Concepts on the Role of Nuclear Factor Erythroid-Derived 2-Like 2 (Nrf2) in Age-Related Diseases. Int. J. Mol. Sci..

[39] Carrington E.M., Tarlinton D.M., Gray D.H., Huntington N.D., Zhan Y., Lew A.M. (2017). The Life and Death of Immune Cell Types: The Role of BCL-2 Anti-Apoptotic Molecules. Immunol. Cell Biol..

[40] Salehi F., Behboudi H., Kavoosi G., Ardestani S.K. (2018). Oxidative DNA Damage Induced by ROS-Modulating Agents with the Ability to Target DNA: A Comparison of the Biological Characteristics of Citrus Pectin and Apple Pectin. Sci. Rep..

[41] Feng D., Huang H., Yang Y., Yan T., Jin Y., Cheng X., Cui L. (2015). Ameliorative Effects of N-Acetylcysteine on Fluoride-Induced Oxidative Stress and DNA Damage in Male Rats’ Testis. Mutat. Res. Toxicol. Environ. Mutagen..

[42] El-Kashlan A.M., Nooh M.M., Hassan W.A., Rizk S.M. (2015). Therapeutic Potential of Date Palm Pollen for Testicular Dysfunction Induced by Thyroid Disorders in Male Rats. PLoS ONE.

[43] Wang J.L., Zhang H.J., Wang H.L., Wang J.W., Gou P.H., Ye Z.H., Wang Y.L. (2015). Influence of Hypothyroidism on Oxidative Stress, c-Fos Expression, Cell Cycle, and Apoptosis in Rats Testes. Toxicol. Environ. Chem..

[44] Ahmad R., Hussain A., Ahsan H. (2019). Peroxynitrite: Cellular Pathology and Implications in Autoimmunity. J. Immunoass. Immunochem..

[45] Bauer G. (2015). Increasing the Endogenous NO Level Causes Catalase Inactivation and Reactivation of Intercellular Apoptosis Signaling Specifically in Tumor Cells. Redox Biol..

[46] Sivrikaya A., Kolayli S., Kucuk M., Aliyazicioglu R. (2009). In Vitro Effects of Peroxynitrite Treatment on Fish Liver Catalase Activity. J. Enzym. Inhib. Med. Chem..

[47] Heinzelmanna S., Bauer G. (2010). Multiple Protective Functions of Catalase against Intercellular Apoptosis-Inducing ROS Signaling of Human Tumor Cells. Biol. Chem..

[48] Chang X.R., Yao Y.L., Wang D., Ma H.T., Gou P.H., Li C.Y., Wang J.L. (2019). Influence of Hypothyroidism on Testicular Mitochondrial Oxidative Stress by Activating the P38 Mitogen-Activated Protein Kinase and c-Jun NH 2 -Terminal Kinase Signaling Pathways in Rats. Hum. Exp. Toxicol..

[49] Bhattarai K.R., Chaudhary M., Kim H.-R., Chae H.-J. (2020). Endoplasmic Reticulum (ER) Stress Response Failure in Diseases. Trends Cell Biol..

[50] Wu J., Kaufman R.J. (2006). From Acute ER Stress to Physiological Roles of the Unfolded Protein Response. Cell Death Differ..

[51] Zhang L.H., Zhang X. (2010). Roles of GRP78 in Physiology and Cancer. J. Cell. Biochem..

[52] Ham J., Lim W., You S., Song G. (2020). Butylated Hydroxyanisole Induces Testicular Dysfunction in Mouse Testis Cells by Dysregulating Calcium Homeostasis and Stimulating Endoplasmic Reticulum Stress. Sci. Total Environ..

[53] Ham J., You S., Lim W., Song G. (2020). Etoxazole Induces Testicular Malfunction in Mice by Dysregulating Mitochondrial Function and Calcium Homeostasis. Environ. Pollut..

[54] Hillary R.F., Fitzgerald U. (2018). A Lifetime of Stress: ATF6 in Development and Homeostasis. J. Biomed. Sci..

[55] Yu R., Chen X., Zhu X., He B., Lu C., Liu Y., Xu X., Wu X. (2022). ATF6 Deficiency Damages the Development of Spermatogenesis in Male Atf6 Knockout Mice. Andrologia.

[56] Yachie A. (2021). Heme Oxygenase-1 Deficiency and Oxidative Stress: A Review of 9 Independent Human Cases and Animal Models. Int. J. Mol. Sci..

[57] Campbell N.K., Fitzgerald H.K., Dunne A. (2021). Regulation of Inflammation by the Antioxidant Haem Oxygenase 1. Nat. Rev. Immunol..

[58] Poss K.D., Tonegawa S. (1997). Heme Oxygenase 1 Is Required for Mammalian Iron Reutilization. Proc. Natl. Acad. Sci. USA.

[59] Yao H., Peterson A.L., Li J., Xu H., Dennery P.A. (2020). Heme Oxygenase 1 and 2 Differentially Regulate Glucose Metabolism and Adipose Tissue Mitochondrial Respiration: Implications for Metabolic Dysregulation. Int. J. Mol. Sci..

[60] Hegab I.I., El-Latif R.N.A., El-Horony H.E. (2019). Targeting Heme Oxygenase-1 in Hypothyroidism Induced Reproductive Dysfunction in Adult Male Rats. Med. J. Cairo Univ..

[61] Yao Y., Chang X., Wang D., Ma H., Wang H., Zhang H., Li C., Wang J. (2018). Roles of ERK1/2 and PI3K/AKT Signaling Pathways in Mitochondria-Mediated Apoptosis in Testes of Hypothyroid Rats. Toxicol. Res..

[62] Huang Y., Guo Y., Huang L., Fang Y., Li D., Liu R., Lu Q., Ren R., Tang L., Lian L. (2021). Kisspeptin-54 Attenuates Oxidative Stress and Neuronal Apoptosis in Early Brain Injury after Subarachnoid Hemorrhage in Rats via GPR54/ARRB2/AKT/GSK3β Signaling Pathway. Free Radic. Biol. Med..

[63] Ilie M., Khambata-Ford S., Copie-Bergman C., Huang L., Juco J., Hofman V., Hofman P. (2017). Correction: Use of the 22C3 Anti-PD-L1 Antibody to Determine PD-L1 Expression in Multiple Automated Immunohistochemistry Platforms. PLoS ONE.

[64] Silva J.F., Ocarino N.M., Serakides R. (2014). Maternal Thyroid Dysfunction Affects Placental Profile of Inflammatory Mediators and the Intrauterine Trophoblast Migration Kinetics. Reproduction.

[65] Livak K.J., Schmittgen T.D. (2001). Analysis of Relative Gene Expression Data Using Real-Time Quantitative PCR and the 2^−ΔΔCT^ Method. Methods.

[66] Bradford M.M. (1976). A Rapid and Sensitive Method for the Quantitation of Microgram Quantities of Protein Utilizing the Principle of Protein-Dye Binding. Anal. Biochem..

[67] Marklund S., Marklund G. (1974). Involvement of the Superoxide Anion Radical in the Autoxidation of Pyrogallol and a Convenient Assay for Superoxide Dismutase. Eur. J. Biochem..

[68] Aebi H. (1984). Catalase in Vitro. Methods Enzymol..

[69] Oliveira K.M., Binda N.S., Lavor M.S.L., Silva C.M.O., Rosado I.R., Gabellini E.L.A., Da Silva J.F., Oliveira C.M., Melo M.M., Gomez M.V. (2018). Conotoxin MVIIA Improves Cell Viability and Antioxidant System after Spinal Cord Injury in Rats. PLoS ONE.

[70] Borges S.C., Tironi L.M.T., da Silva L.M., Buttow N.C. (2020). Curcumin Protects Remote Organs against Injury That Is Caused by Intestinal Ischemia and Reperfusion. Acta Sci. Biol. Sci..

[71] Ferreira G.F., de Matos Baltazar L., Santos J.R.A., Monteiro A.S., de Oliveira Fraga L.A., Resende-Stoianoff M.A., Santos D.A. (2013). The Role of Oxidative and Nitrosative Bursts Caused by Azoles and Amphotericin B against the Fungal Pathogen Cryptococcus Gattii. J. Antimicrob. Chemother..

[72] Silva J.F., Ocarino N.M., Vieira A.L.S., Nascimento E.F., Serakides R. (2013). Effects of Hypo-and Hyperthyroidism on Proliferation, Angiogenesis, Apoptosis and Expression of COX-2 in the Corpus Luteum of Female Rats. Reprod. Domest. Anim..

